# Dietary Intake of Folate and Assessment of the Folate Deficiency Prevalence in Slovenia Using Serum Biomarkers

**DOI:** 10.3390/nu13113860

**Published:** 2021-10-28

**Authors:** Igor Pravst, Živa Lavriša, Maša Hribar, Hristo Hristov, Naska Kvarantan, Barbara Koroušić Seljak, Matej Gregorič, Urška Blaznik, Nadan Gregorič, Katja Zaletel, Adrijana Oblak, Joško Osredkar, Katja Žmitek, Anita Kušar

**Affiliations:** 1 Nutrition Institute, Tržaška cesta 40, SI-1000 Ljubljana, Slovenia; ziva.lavrisa@nutris.org (Ž.L.); masa.hribar@nutris.org (M.H.); hristo.hristov@nutris.org (H.H.); naska.kvarantan@gmail.com (N.K.); katja.zmitek@vist.si (K.Ž.); anita.kusar@nutris.org (A.K.); 2 Biotechnical Faculty, University of Ljubljana, Jamnikarjeva 101, SI-1000 Ljubljana, Slovenia; 3 VIST–Higher School of Applied Sciences, Gerbičeva cesta 51A, SI-1000 Ljubljana, Slovenia; 4 Division of Human Nutrition and Health, Wageningen University & Research, NL-6708 Wageningen, The Netherlands; 5 Computer Systems Department, Jožef Stefan Institute, SI-1000 Ljubljana, Slovenia; barbara.korousic@ijs.si; 6 National Institute of Public Health, Trubarjeva 2, SI-1000 Ljubljana, Slovenia; matej.gregoric@nijz.si (M.G.); urska.blaznik@nijz.si (U.B.); 7 University Medical Centre Ljubljana, Zaloška cesta 7, SI-1000 Ljubljana, Slovenia; nadan.gregoric@kclj.si (N.G.); katja.zaletel@kclj.si (K.Z.); adrijana.oblak@kclj.si (A.O.); josko.osredkar@kclj.si (J.O.); 8 Faculty of Medicine, University of Ljubljana, Vrazov trg 2, SI-1000 Ljubljana, Slovenia; 9 Faculty of Pharmacy, University of Ljubljana, Aškerčeva cesta 7, SI-1000 Ljubljana, Slovenia

**Keywords:** folate, folic acid, folate intake, folate deficiency, homocysteine, Slovenia

## Abstract

Folate deficiency is associated with various health issues, including anemia, cardiovascular disease, and birth defects. Low folate intake and suboptimal folate status were found in several countries; however, this topic has not yet been investigated in Slovenia. Dietary folate intake and serum folate status were investigated through the nationally representative food consumption study SI.Menu/Nutrihealth. Folate intake was estimated using a sample of *N* = 1248 subjects aged 10–74 years, stratified in three age groups (adolescents, adults, elderly population), through two 24 h-dietary recalls and food propensity questionnaire. Data on serum folate and homocysteine was available for 280 participants. Very low folate intake (<300 µg/day) was observed in 59% of adolescents, 58% of adults and 68% of elderlies, and only about 12% achieved the WHO recommended level of 400 µg/day. Major dietary contributors were vegetables and fruit, and cereal products. Living environment, education, employment status and BMI were linked with low folate intake in adults; BMI, and sex in adolescents; and sex in elderlies. Considering low serum folate (<7 nmol/L) and high serum homocysteine (>15 nmol/L), folate deficiency was found in 7.6 and 10.5% in adults and elderlies, respectively. Additional public health strategies should be employed to promote the consumption of folate-rich foods. With current folate intakes, supplementation with folic acid is relevant especially in specific vulnerable populations, particularly in women planning and during pregnancy.

## 1. Introduction

Folate is one of the water-soluble B-vitamins, which cannot be synthesized in the human body. Folate is an essential micronutrient required for the synthesis of both RNA and DNA, for cell division, growth and development [1]. These processes are regulated through many metabolic processes, which can be affected by the lack of folate. Insufficient dietary intake of folate and subsequent deficiency has been associated with various health issues, such as megaloblastic anaemia and cardiovascular disease [2]. Important role of folate in pregnancy is also well established [3]; as adequate folate intake during periconceptional period and the first trimester of pregnancy helps to prevent neural tube defects and other adverse birth outcomes [4].

Diets low in fresh fruits, leafy green vegetables, unrefined grains, and legumes have been linked with folate deficiency [5]. This can occur especially in populations where dietary intake relies more extensively on processed foods, while intake of fresh fruits, vegetables and legumes is often insufficient. On the other hand, regular consumption of such folate-rich foods has been shown as a protective measure [6]. Besides nutrition, lactation and alcoholism are among major factors, contributing to folate deficiency [5,6]. Folate status can be also affected by methylenetetrahydrofolate reductase gene mutation [7] and various diseases [8]. It should be noted that in foods folate is commonly bound to protein or carbohydrate food matrices [9,10], which limit its bioavailability. Losses of folate from foods can occur during cooking, particularly during heating and oxidation [11]. On the other hand, grinding of foods can help release more folates [12] and ascorbic acid improves folate stability [13]. Foods can be also enriched with folate during manufacturing. In the European Union, folic acid (pteroylmonoglutamic acid) and calcium-L-methylfolate are allowed sources of folate for voluntarily enrichment of foods [14], while (6S)-5-methyltetrahydrofolic acid derivatives are also allowed in food supplements [15]. Bioavailability of folate added to such products is generally higher [16] than in foods, where folate is bound to cell structures.

Considering that folate status is affected by such a wide range of parameters, dietary folate intake alone cannot be used to identify those at risk for folate deficiency. Different biological markers are therefore used for this purpose. Most commonly used is serum folate level [17]. Considering that serum folate levels increase up to 2 h after ingestion of folate (followed by rapid decline), it is recommended this biomarker is measured under fasted conditions [18]. With consideration of macrocytic anaemia as a haematological indicator, serum folate cut-off level of 14 nmol/L (6 ng/mL) is typically used as indicator of possible folate deficiency, while levels below 7 nmol/L (3 ng/mL) are interpreted as folate deficiency [19]. Additionally, elevated serum homocysteine is considered as crucial metabolic indicator of folate deficiency [17], with upper reference range limit between 10–19 μmol/L [17], most typically at 15 μmol/L [20,21]. Independently, hyperhomocysteinemia is also presenting an important risk factor of atherosclerosis and other cardiovascular disease, including arterial endothelial dysfunction and thromboembolism [22,23,24]. Besides serum folate and homocysteine, there are several other biomarkers of folate deficiency. Very valuable biomarker is folate concentration in red cells [25], which is not affected by short-term dietary interferences. Examples of other possible biomarkers are 5-methyltetrahydrofolate in cerebrospinal fluid, urinary folate, etc. [17].

In this paper term “folate” is used for all active forms of this vitamin, while folic acid is used specifically for pteroylmonoglutamic acid. The latter one is also one of the authorised forms of folate for use in enriched foods, food supplements and medicines. Speaking of requirements, these are set for folate equivalents and refer to all the natural and synthetic forms of folate. According to US Institute of Medicine (IOM) [26] and World Health Organisation (WHO) [27], estimated average requirements (EARs) of folate for adolescents (10–18 years) and adults/elderly (above 19 years) is 330 and 320 µg, respectively, while its recommended nutrient intakes (RNI) is 400 µg. European Food Safety Authority (EFSA) set population reference intake at 330 µg/day and average requirement (AR) at 250 µg/day [28]. According to D-A-CH recommendations (D, A and CH assign Germany, Austria and Switzerland, where these recommendations were prepared), which are also implemented in Slovenia [29], in year 2013 the reference daily intake for folate equivalents in adults was lowered from 400 µg [30] to 300 µg [29,31]. While there is no nationally representative data available on the actual folate intake in the general adult population, nor on the prevalence of folate deficiency, low folate intake/status was reported in specific vulnerable groups [32,33]. On the other hand, Rippin et. all reviewed data available from other European countries and reported that only a few populations met recommended folate intakes [34]. Pooled mean folate intake was 268 µg and 318 µg in women and men, respectively. Due to a public health concern of folate deficiency, some countries introduced the mandatory fortification of certain foods with folate [35], which resulted in increased folate intakes and improved folate status [5,36]. With consideration of possible adverse outcomes of excess vitamin intakes, changes in fortification policies should be planned very carefully [37]. To assure adequate folate intake, in Slovenia supplementation with folic acid is currently recommended to pregnant women and those planning pregnancy [38], however, there are no requirements or recommendations regarding the general population or mandatory food fortification. According to research, folate is also one of the micronutrients which can be critical in the elderly [39], as its insufficiency can cause various health problems, including cognitive performance, so supplementation could be also considered [40]. On the other hand, for example, in athletes, higher plasma folate combined with training could help to keep homocysteine levels at optimum [41]. There are general EU rules enabling voluntarily enrichment of foods with vitamins [42], but foods are quite rarely enriched with folate. Reported prevalence of labelled folate content on prepacked foods in Slovenia is 3% [43], but higher prevalence was observed on processed breakfast cereals (34%) and functional drinks (27%).

The objective of the present study was to estimate dietary folate intake and to determine the prevalence of folate deficiency in different Slovenian population groups. We exploited the data collected in the nationally representative food consumption study on adolescents, adults and elderly population (SI.Menu study), upgraded with collection of biological biomarkers on sub-sample of adults and elderlies (Nutrihealth study). Secondary objectives were to determine main sources of folate in peoples’ diets, and to investigate determinants affecting low folate intake and folate deficiency.

## 2. Material and Methods

### 2.1. Study Design and Subjects

Data for estimation of folate intake was obtained from a cross-sectional national food consumption study (SI.Menu), which was conducted using cross-sectional approach in period from March 2017 to April 2018. Study was conducted in line with the EFSA’s Guidance on European Union Menu Methodology [44]. More details on the methodology of the SI.Menu study was published previously [45]. Altogether, *N* = 2280 participants were randomly selected from Central Register of Population of Slovenia, with separate quotas for adolescent (10–17 years), adult (18–64 years), and elderly population (65–74 years), with 62% response rate. Exclusion of subjects with missing data and under/over-reporters (*N* = 97) is previously described [45,46]. Final SI.Menu study sample included *N* = 1248 subjects (468 adolescents, 364 adults and 416 elderlies).

Data for estimation of folate deficiency was obtained within Nutrihealth study, which was detailly described elsewhere [47]. Nutrihealth was conducted as an extension of the SI.Menu study. In short—after finished participation in the above described SI.Menu study, a sub-sample of adults and elderlies from SI.Menu study was invited to participate with blood and spot urine sample. A final sample of participants who provided samples for biomarker analysis included *N* = 280 participants; for these serum folate and homocysteine was determined (125 and 155 adults and elderlies, respectively).

### 2.2. SI.Menu Study Data Collection and Analyses

In the SI.Menu study, food consumption data and corresponding metadata was collected using General Questionnaire for socio-demographic and socio-economic data, and Food Propensity Questionnaire (FPQ) for collecting consumption frequency of key foods from selected food categories [48]. Both questionnaires were completed during personal visit, based on participants’ answers. At same visit, anthropometric data, body height (m) and body mass (kg) were also collected. For adults body mass index (BMI) (kg/m^2^) was interpreted using overweight cut-off point at 25 kg/m^2^, while sex/age adjusted cut-off points (>1 standard deviation (SD)) were applied for adolescents [49,50]. Participant’s physical activity was estimated using International Physical Activity Questionnaire (IPAQ) score [51]. General Questionnaire also contained questions which were used to identify participants’ smoking status (non-smoker; smoker/occasional smoker/ex-smoker), medical (no diet, medical/weight loss diet) or other diets (no diet; vegetarian/vegan). Demographic characteristics was also collected and used for analyses coding: residential area (rural, intermediate, urban), education level (university degree, no university degree), self-reported financial status (below/above average, using monthly income cut-off at 1300€), employment status (employed/unemployed, student, retired).

Additionally, participant’s dietary habits were investigated with two non-consecutive 24-h dietary recalls, executed 1–3 weeks apart (71% and 29% of the recalls were performed on workdays and weekends, respectively). Nationally developed picture book was used to support estimation of portion sizes. This tool was developed specifically for the conduction of the national food consumption study, and was composed of 46 pictures of foods, each presented in six portions [52].

### 2.3. Assessment of Dietary Folate Intake

Available food consumption data (24-h recalls) was analysed using the Open Platform for Clinical Nutrition (OPEN) [53], which contain Slovenian food composition database. This database consists of nutritional composition data for both generic and branded foods and provides traditional recipes of foods which are frequently consumed in Slovenia. Altogether, *N* = 2377 different foods were extracted from the SI.Menu study consumption dataset. For foods without data about folate content in the OPEN tool, missing information was taken from other sources, primarily from the FINELI [54] and USDA [55] food composition databases. Manual food-matching was done by trained nutritionist. Altogether, 89.5% of the reported foods were determined to be source of dietary folate. To enable estimation of daily folate intake with the use of Multiple Source Method (see Section 2.4), each reported food was assigned into one of pre-defined food categories, with consideration of FPQ. For the reporting purpose, all foods were additionally assigned into food categories, adapted by Global Food Monitoring Initiative [56]. Such categorisation was applied to describe the relative contribution of food categories in population usual daily dietary folate intake.

### 2.4. Serum Folate and Homocysteine Concentration

Folate and homocysteine concentrations were measured in serum of the participants of the Nutrihealth study at the University Medical Center (Ljubljana, Slovenia). Folate was analysed at the Department of Nuclear Medicine, while homocysteine was analysed at the Institute of clinical chemistry and biochemistry.

Folate was measured in serum with the chemiluminescence immunoassay determined on an Immulite 2000 XPi analyzer (Siemens Healthineers, Gwynedd, UK). Performance characteristics for the assay are as follows: Limit of detection is 1.8 nmol/L, linearity of the assay is in the range from 2.3 to 54 nmol/L with recovery range 96% to 106%. The intra-assay and inter-assay coefficients of variation range from 4.2% to 5.0% and from 4.6% to 5.5%, respectively. Cross-reactivity of the assay showed 0.9% cross-reactivity with 100 µg/L of Methotrexate. A cut-off of 7 nmol/L was used to identify subjects with low serum folate level. Additionally, serum concentration lower than 10 nmol/L was considered as marginally low folate concentration.

Homocysteine was measured using chemiluminescence immunoassay method on an IDS-iSYS analyzer (Immunodiagnostic Systems, Boldon, UK). Literature data showed good correlation (*r* = 0.95–0.99) between immunoassay methods and HPLC method [57]. Criteria for elevated serum homocysteine concentration was set at 15 μmol/L [17,20]. Additionally, more than 10 μmol/L was considered as marginally elevated serum homocysteine concentration.

As recommended [17], a combination of both—low serum folate concentration (<7 nmol/L) and elevated serum homocysteine concentration (>15 μmol/L) was used as criteria for identification of subjects with high risk for folate deficiency. Study protocol did not enable analyses of other parameters, such as red cell folate.

### 2.5. Data Analyses

Data collected in two 24-h recalls and the FPQs was used to estimate usual daily folate intakes, separately for all three age cohorts. Folate intake distributions per age group were adjusted for individual day-to-day variation using Multiple Source Method (MSM) [58]. A similar approach was used in our previous study, investigating vitamin D intake [59]. Age, sex, and BMI were considered as covariates. With the use of MSM we calculated individual daily usual folate intakes [60]. Year 2017 census data was used for population-weighting by iterative proportional fitting [61]. All population-weighted descriptive characteristics are reported separately for all three age groups, males and females. Population weighted mean folate intake was calculated (using SI.Menu study data) in µg/day, and in µg per daily intake of 1000 kcal. Proportion of the population meeting recommended daily folate intake was calculated two thresholds: nationally implemented D-A-CH recommendation [31] 300 µg and IOM/WHO recommendation 400 µg [26,27]. Population-weighted serum folate and homocysteine concentrations was also calculated (using Nutrihealth study data) separately for all three age groups. Prevalence of low serum folate and high serum homocysteine concentration was calculated using two cut-off values (<7 and <10 nmol/L for folate, and >10 and 15 µmol/L for homocysteine). We also calculated population-weighted prevalence of folate deficiency, using criteria of low serum folate (<7 nmol/L) and high serum homocysteine level (>15 μmol/L).

A series of regression analyses were conducted. Linear regression was employed to calculate energy-adjusted mean daily folate intakes, separately for all three cohorts, with consideration of sex, residential area, education level and financial status (only for adults and elderlies); BMI; IPAQ scores; employment status (only for adults); smoking status; and diet type. These parameters were also used in the logistic regression analyses to calculate odd ratios (OR) for meeting daily recommended intake of 300 µg folate. For models with serum folate concentrations, regression analysis was done on combined study sample of adults and elderlies (*N* = 271; 9 subjects were excluded due to missing dietary data). Odd ratios for low serum folate concentration (<7 nmol/L) were therefore calculated also with parameter of age group. Additional parameters in the analyses were sex, residential area, education level, financial status; smoking status, BMI; IPAQ; diet; supplement use, daily folate and energy intake, and serum homocysteine concentration. Similar approach was also used for OR for folate deficiency (serum folate <7 nmol/L and homocysteine >15 μmol/L), with following parameters in the model: age group, sex, residential area, education level, financial status; smoking status, BMI; IPAQ and daily folate energy intake. Again, model was adjusted for daily energy intake.

STATA (version 17.0; StataCorp LLC, Coledge Station, TX, USA) was used for statistical analyses. Statistical significance was set at *p* < 0.05. Means are reported with either a standard deviation (SD) or standard error (SE).

## 3. Results

In Table 1, the demographic characteristics of the SI.Menu sample are described (*N* = 1248). Only few participants (*N* = 9) explicitly reported supplementation with folate, while occasional use of multivitamin food supplements was reported by about a quarter of participants. About one third of adult and elderly SI.Menu participants (34% of adults and 37% of elderlies) were also included to Nutrihealth study, where biological samples (serum) were also collected.

**Figure 1:**
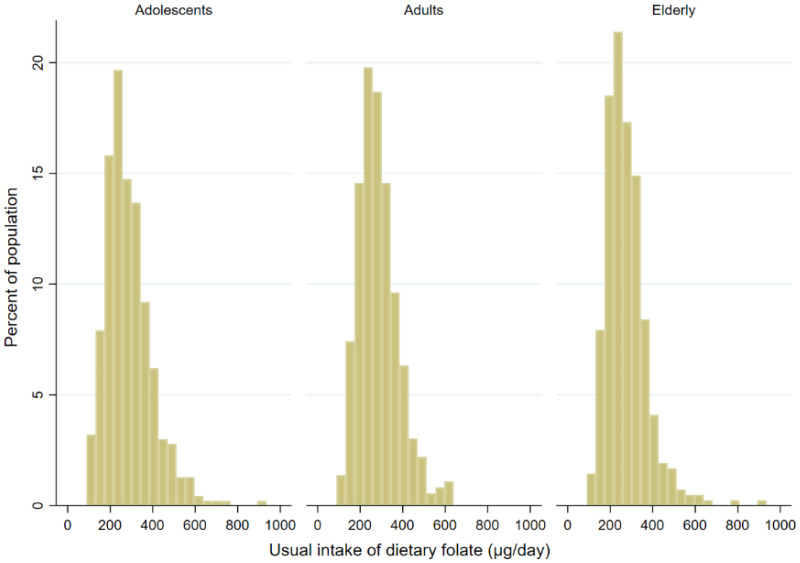
Histograms of estimated usual daily intake of folate for all three age cohorts (adolescents: 10–17 years; Adults: 18–64 years; Elderly: 65–74 years).

Population-weighted descriptive statistics are shown Table 2, while distribution of estimated usual daily intake of folate is presented in histograms in Figure 1. In all age groups mean daily folate intake was below the recommended 300 µg/day, however somewhat higher mean was observed in specific subgroups, e.g., male adolescents and elderly females. The usual mean daily folate intake was quite similar in elderly (295.5 µg/day) and adult population (294.6 µg/day), and somewhat lower in adolescents (289.8 µg/day) (Table 2). The mean daily folate intake was generally lower for women, except in elderly, opposite trend was observed after consideration of daily energy intake. Folate intake calculated per 1000 kcal/day was higher for women in all three population groups. Same trend was also observed in a regression analyses model, when higher adjusted mean daily folate intake was observed in women in all population groups (Supplementary Table S1). Analyses showed sex a significant predictor of mean daily folate intake in elderlies and adolescents. In adolescents and adults, body mass index was also found a significant parameter, with lower folate levels observed in those with overweight/obesity. Additionally, adults with higher education and with employment had higher daily folate intakes. Population-weighted intake data show that about one third of the population met daily D-A-CH recommended folate intake of 300 µg/day; index was lower in the elderly (32.2%) than in adults (41.9%) and adolescents (41.2%). About 12% of each population group met IOM/WHO target value of 400 µg/day. Predictors associate with daily folate intake level (cut-off 300 µg) were determined using logistic regression analyses, with separate model for each population group (Table 3). In both, adolescents and adults, females compared to males had significantly lower odds for meeting recommended daily folate intake level (OR 0.63 and 0.44, respectively). Other factors significantly associate with daily dietary folate intake level were BMI in adolescents (OR 0.65 for overweight/obese) and education in adults (OR 1.93 for higher education). Employment status and residential area were notably associated with the daily dietary folate intake level (*p* = 0.06 and 0.07, respectively), with lowest odds observed for unemployed and those living in rural areas. Similar trends were observed in elderly.

**Figure 2:**
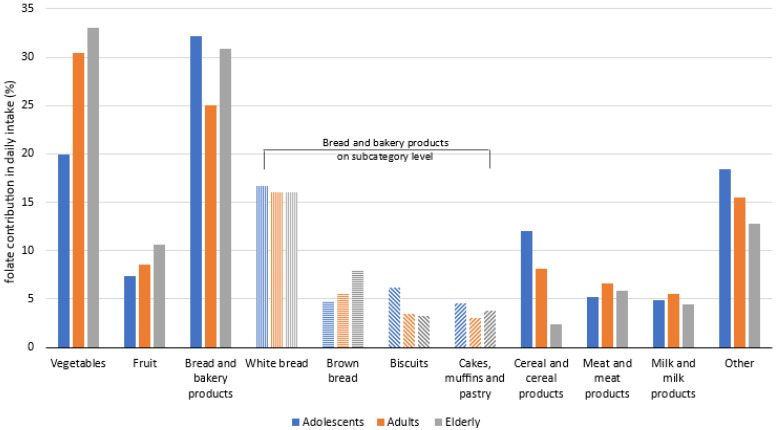
Relative contribution of selected food categories to daily folate intake among all three age cohorts (% of total folate intake; adolescents: 10–17 years; Adults: 18–64 years; Elderly: 65–74 years).

To provide further insights, we investigated relative contribution of different foods to daily folate intakes (Figure 2, Supplementary Table S2). Food categories with the greatest contribution to daily folate intake were fruit and vegetables, bread, bakery products and other cereal products. The latter includes various types of breakfast cereals, which were more important folate contributors in adolescents. Among bread and bakery products, brown bread had higher contribution of folate in elderly than in other population groups. Altogether, most notable difference between groups was observed in vegetables, which contributed 27% folate intake in adolescents, and about 40% in adults and elderlies. Milk, meat, and products of thereof were also shown as notable contributors to folate intake.

**Figure 3:**
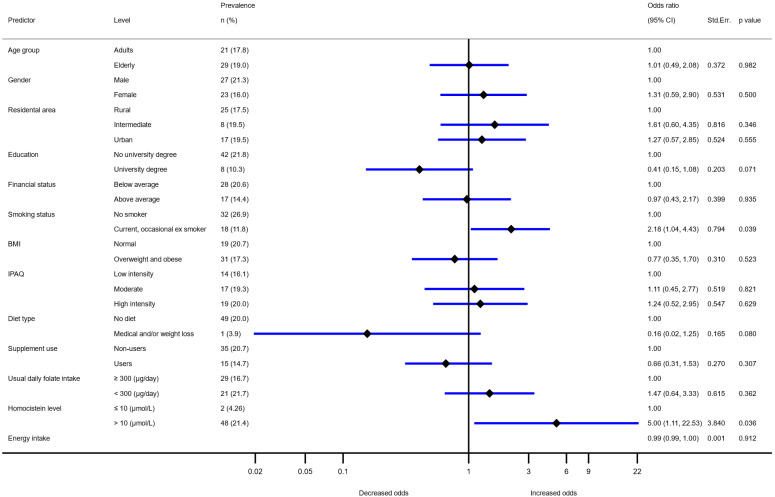
Association between serum folate concentration level (<7 nmol/L) and age, sex, residential area, education, family income, BMI (Body mass index), IPAQ (International Physical Activity Questionnaire) score, diet type, supplementation with multivitamins/folate, daily dietary folate intakes, and homocysteine status. Model adjusted for daily energy intake (*N* = 271).

For adults and elderlies (but not for adolescents) we also investigated folate status with the use of biomarkers, i.e., serum folate and homocysteine concentration (Table 2). Mean population-weighted serum folate level in adults and elderlies was 10.6 and 11.4 nmol/L, respectively. Almost half of adult and elderly population had serum folate levels lower than 10 nmol/L, while very low folate concentrations (<7 mol/L) were observed in 17% adults and 18.5% elderlies (22% in males, and 15% in females). Logistic regression analyses model identified smoking (OR 2.18; 1.04, 4.43, *p* = 0.04) and serum homocysteine levels above 10 μmol/L (OR 5.0; 1.1, 22.5; *p* = 0.04) as predictors for folate deficiency (Figure 3). Education and diet were also found close to significant (*p* = 0.07 and 0.08, respectively), with lowest OR in those with higher education and on medical diet.

Interestingly, use of folate/multivitamin supplements and daily folate intake were not found significant predictors. However, we observed expected trend of lowest OR in supplement users and those with adequate daily folate intake.

Further, we investigated the prevalence of those with folate deficiency, with consideration of both low serum folate (<7 nmol/L) and high homocysteine (>15 µmol/L) concentration. Notably higher population-weighted folate deficiency prevalence was observed in elderlies (10.1%; 95%CI: 6.3–16.0), in comparison to other adults (6.9%; 95% CI: 3.5–13.0%). In both population groups, notably higher prevalence was observed in men (9.7% in adults and 14.5% in elderlies). Low number of subjects with identified folate deficiency limited our ability for identification of significant deficiency predictors, but the observed trends are comparable with the low folate levels. Notably higher prevalence of folate deficiency was observed in elderlies, males, smokers, living in rural areas, with lower education and lower income (Supplementary Table S3).

## 4. Discussion

The present study is the first one evaluating folate intake and status in the Slovenian general population. With consideration of both—daily folate intakes, and biomarkers of folate status, we identified risks that are comparable with several other countries within and outside Europe [5]. Looking into folate intakes, the observed current situation is comparable to situation in the USA before the introduction of food fortification in the year 1998, where folate intake in the population was below the recommended requirement in 49% of the population [36,62]. Our data show a somewhat higher risk for low folate intake in elderlies, and a similar trend was also observed with folate biomarkers. Folate deficiency (low serum folate (<7 nmol/L) and high serum homocysteine (>15 µmol/L) concentration) was found in 7% of adults and 10% of elderly population. It should be noted that mean daily folate intake in elderlies can be deceiving, despite being the highest among population groups (Table 2), we observed higher inter-individual variability, in comparison to adults and adolescents, meaning that on one hand there are individuals with low folate intakes and on the other, those with high folate intakes. As seen from Figure 1, among elderly population there is a high proportion of those with usual folate intakes below 300 µg/day and on the other hand we also identified higher proportion of subjects with above average folate intakes, which affected estimation of mean daily folate intake. This is also evident from data on the prevalence of adequate folate intake. Elderlies were population group with the lowest proportion (32%) of individuals meeting D-A-CH recommendation for daily folate intake (300 µg). Folate related risks in the elderly population were also highlighted in other studies [63,64,65,66].

Available data show that dietary folate intake in European countries varies, with no clear gradient between countries’ geographical regions. With the exception of some populations in Lithuania, Ireland, and Turkey, folate intake in EU countries is typically well below the WHO/NIH recommendation of 400 µg/day, and mostly even lower than 300 µg/day [34]. Particularly low folate intakes were reported in some Nordic countries [34,67]. In our study, except for the elderly population, males had significantly higher folate intakes, which was also observed in some other studies [68,69,70]. However, after adjustment for energy intake, in all three age cohorts, women had a higher folate intake (per 1000 kcal) than men (Table 2). A similar observation was noted in a study on a Finnish population, after folate intake was adjusted for daily energy intake [71]. Adjusting folate intake for daily energy intake provides very interesting insights. While men tend to consume a higher quantity of food compared to women, which can result in higher intakes of energy and certain (macro)nutrients, the nutritional density of such diet is clearly lower, at least for folate. Adjusting for energy intake can be an important factor, when studying sex differences in dietary intakes [72,73]. Because same daily recommended folate intake was used for both males and females, the regression analyses for meeting such intake (Table 3) was not adjusted for daily energy intakes. However, energy adjustment was used in the regression analyses on mean folate intakes (Supplementary Table S3).

Lower energy-adjusted mean dietary folate intakes were associated with certain sociodemographic and lifestyle factors, especially in adults. In the elderly population, besides sex, other investigated parameters did not significantly influence daily folate intake (Supplementary Table S1). In the adult population, significant determinants of daily folate intake were residential area, education, employment status and BMI, which was also significant predictor in adolescents. Adults living in rural areas had significantly lower folate intake compared to others, and similar was evident for unemployed, retired, as well as for those less educated. The influence of some of these factors was reported elsewhere as well, with those living in rural areas [74], lower level of education [75] and higher BMI [76] being at higher risk for poorer nutrition, including lower dietary folate intake. Besides, lower economic status is often associated with higher BMI [77,78,79], which even strengthens the link between factors leading to nutritionally inadequate diets. This implies that such populations are more vulnerable in terms of nutrition, which was seen also in Slovenia [79]. They tend to consume more processed foods and less nutritiously rich foods, such as fruit, vegetables, unrefined grains, which can result in inadequate nutrient intake. Those with lower incomes are also considerably more affected by food prices [80]; as various processed foods are often available for a lower price than whole fresh foods, this can pose an additional threat to such vulnerable populations [81]. Our observations support calls that public health policies should also consider economic barriers in these fragile population groups because an abundance of cheaper processed foods of poor nutritional density is making nutritious health diets more and more challenging [82].

We observed that in all investigated population groups the consumption of “Bread and bakery products” has high impact on folate intake, similar to “Vegetables”. This can be explained by the fact, that in Slovenia bread is consumed on daily basis, sometimes even more than once per day. On the other hand, some vegetables are very rich in folate, so compared to bread, the higher folate intake can be achieved with smaller amounts of such foods consumed. This explains high folate intake from vegetables, although according to national food consumption data, the daily intake of vegetables intake in the population is generally still below recommendations [79]. Our study indicates that more regular consumption of folate-rich foods such as fruits, vegetables and wholegrains would be helpful to improve folate intake in the population. Vegetables, fruit, bread, and other cereal products were also seen as the major dietary folate contributors in other studies [71,76,83]. The folate contribution from daily diet is the greatest from fruits and vegetables in the elderly, where these food categories present more than 40% of total daily dietary folate intake. Additionally, we should also note that number of participants following vegan or vegetarian diet was too low for reliable insights. Interestingly, in adolescents, the contribution of folate from fruits and vegetables was below 30%, but a higher contribution was observed in bread and bakery products, particularly in white bread. They also get more folate from other cereal products, including (fortified)breakfast cereals, which are more popular in adolescents than in other population groups. These results are in line with the observations that consumption of such processed/refined foods is more common in the younger population. Milk, dairy, and meat products were also identified as notable sources of folate. The strategies to encourage adolescents into consuming more unprocessed foods, such as fruit, vegetables, and wholemeal foods (including bread) could help them to achieve higher folate intake, as currently their usual daily folate intake is the lowest among populations and is often achieved from foods which should not be consumed in abundance (white bread, biscuits, etc.). This is also supported with the results for folate density in diets of adolescents, which is notably lower in adolescents (128 µg/1000 kcal), than in adults and elderlies (138 and 141 µg/1000 kcal, respectively).

We also investigated biomarkers for folate deficiency. Interestingly, dietary intake of folate was not found among the strongest predictors of low serum folate levels. This phenomenon has been previously reported [84], and could be explained also with notable differences in bioavailability of folate from different food matrixes [85,86]. Significantly increased odds for low serum folate were observed in smokers, which is comparable with literature data [87,88]. Furthermore, the trends, although not significant, but corresponding to the literature data [89,90] also show a higher likelihood for low folate or folate deficiency in elderlies, males, with lower education or financial status. Interestingly, similar trends were observed to impact dietary folate intakes. As expected, higher serum homocysteine concentration was found a significant predictor of lower folate status. However, homocysteine levels can be affected by several different lifestyle factors, as well as personal characteristics, including age and sex [91,92]. The link between serum folate and homocysteine should be interpreted with caution, as not every increase in serum homocysteine is caused by low folate status [93]. However, low serum folate combined with elevated serum homocysteine is considered a better indicator for folate deficiency than serum folate alone [17].

With consideration of folate deficiency rates observed in our study, particular attention is required in the elderlies, although we should be aware that the deficiency, to some extent, is also present in a general adult population. Nutrihealth study was designed in a way, to also provide deeper insights into the vulnerable population of elderlies. In elderlies, low folate status is associated with adverse health effects, such as psychiatric and cognitive problems [63,66,94]. Timely intervention with encouragement of consuming folate-rich foods and possible folic acid supplementation should be considered in order to prevent negative outcomes, related to low folate intake and status in this vulnerable population.

However, women of childbearing age are also considered a very important population group, where sufficient folate status is crucial [95,96], but they were not investigated as a separate sample. It was recognized that the risk for neural tube defects is halved if the standard diet is supplemented with 400 μg of folic acid [96]. Therefore, women are recommended to take folic acid at least one month before to at least three months after conception. In many countries, less than 50% of women follow these recommendations [97,98]. Furthermore, pregnancies are not always planned, and some researchers note that fortification of foods with folic acid could substantially lower risks for neural tube defects in Europe [99]. In our study, adult women were the population with the lowest prevalence of folate deficiency, but we should note that the prevalence of vitamin supplements use was also the highest in this group. Altogether, the diet was supplemented with folate/multivitamins by 51% of adult females and 26% of males, but the study design did not enable further insights into the use of food supplements (i.e., to investigate typical daily dosages etc.). Although mean folate intake (270 µg/day), suffices the folate AR set by EFSA (250 µg/day), only 32% of this population met recommended daily folate intake of 300 µg (while only 8% met WHO/NIH recommendation of 400 µg folate), supplementation with folic acid is extremely important for women in childbearing age to reduce risks for neural tube defects in offsprings. Suboptimal folate intakes of women of childbearing age were reported elsewhere as well [100,101,102]. It should be also mentioned that the incidence of neural tube defects in Slovenia did not improve after voluntarily supplementation was advised to women who plan pregnancy [103], meaning that additional efforts are needed to achieve sufficient folate intake in this vulnerable population. A possible approach would be the introduction of reimbursed prescription of folate medicines to all pregnant women and those planning pregnancy, but such approach would also be notably affected by compliance rates and would not provide for those with unplanned pregnancies.

This study highlighted that non-optimal folate intake could be addressed by promoting the consumption of folate-rich foods, particularly fruit, vegetables, unrefined grains, and legumes. It has been previously shown that by a 10% increase in folate intake from foods, a 6% increase in serum folate could be achieved [104]. Such a shift in people’s diets would also provide other benefits, for example, higher intake of dietary fibre, which is also not sufficient in the Slovenian population [105]. As adolescents tend to consume more unrefined grain products, processed food and less fruit and vegetables, innovative educational approaches and careful meal planning in schools could help to improve the situation. There are some inequalities observed considering demographic factors. Public health strategies should therefore focus on promoting and assuring the availability of healthy food for everyone as well as offering knowledge on healthy nutrition, with particular focus to vulnerable groups of the population, such as those less educated, unemployed, living in rural places, elderly, and those with higher BMI. These fragile population groups were also identified to be at risk for other nutrition and health-related factors [79].

Another option for increasing folate intakes in fortification of certain foods with folate, as practiced in some countries, such as USA [106], Canada [107] and Chile [108]. Such practice resulted not only in improvement of the folate status but also in notably lower prevalence of neural tube defects [5]. The latter was also the key reason for the decision to introduce mandatory fortification of non-wholemeal wheat flour in the United Kingdom very recently [109]; they expect the intervention will help to prevent neural tube defects for about 20% (around 200 per year). Interestingly, folate food fortification in the USA led to a higher mean increase in folate intake than initially predicted [36]. After fortification, the mean dietary folate intake in the population increased from 275 to 351 µg/day, while the mean serum folate concentration increased from 11.4 to 26.9 nmol/L [110]. To achieve maximal reductions in serum homocysteine concentrations by folate supplementation, daily doses of more than 800 µg folate is typically required [93]. Recently, a collaborative study performed by the European Commission Joint Research Centre (JCR) and the European network of population-based registries for the epidemiological surveillance of congenital anomalies (EUROCAT) highlighted that folic acid fortification of grain products could contribute to prevention of at least 1000 birth anomalies in the EU every year, despite the fact that folic acid supplementation is already advised, but obviously not always efficiently implemented [111]. However, in many countries, including in the EU, we have no practice of mandatory fortification of foods with folate. This is partly because of the limited evidence on the expected additional health benefits in clinical trials, due to feared health risks and because of the issue of freedom of choice [5], and also because only some specific populations could benefit from high(er) folate intake [37]. Requirements for folate intake are complex and affected by various factors. In foods, folate can be found in various forms, which have different properties, functionalities and bioavailability, while some individual (including genetic) factors should be also appraised [112]. This makes the decision for mandatory fortification even more challenging, however, any other approach might be very limited in assuring sufficient folate intake in future mothers, at least in cases of unplanned pregnancies.

Major strengths of this study are that folate intakes were estimated using food consumption data, collected in a nationally representative population of adolescents, adults, and elderlies, and that for sub-sample of adults and elderlies we were also able to investigate serum biomarkers of folate deficiency. Study strength is also that SI.Menu food consumption study was done with internationally harmonised EU Menu methodology, employing both 24 h recalls and an FPQ. However, some study limitations also need to be mentioned. Considering that folate content was not included in some foods in the OPEN food composition dataset, international food composition databases were also used. Also, the cooking and processing of foods can result in folate losses, which was not considered in the calculation of the intakes. Different forms of folate with different bioavailability were also not considered. We should also mention that this study estimated folate intake with foods, and not with dietary supplements or medicines. While in the SI.Menu study we tracked the use of multivitamin and folate supplements, the collected data did not allow us to estimate folate intakes from these sources. Folate was very rarely supplemented alone; reported use of multivitamin supplements was highest in adults (37%), particularly in women (51%). Considering that supplementation can present an important additional source of folate, this topic should be addressed in further studies. This is even more relevant in the context of the COVID-19 pandemic, which affected people’s lifestyle and behaviours [82], and the use of dietary supplements [83]. We should also mention that while serum folate and homocysteine concentrations are considered as key biomarkers for the identification of folate deficiency [17,18,19], red blood cell folate would provide a better indication for long-term folate status [25,113]. Also, while we used a standard medical diagnostic method for measuring folate in human serum, the chemiluminescence immunoassay is sensitive to very high biotin concentrations. Considering that in serum with biotin levels up to 1500 µg/L such a measurement error is lower than 10%, we did not use control for serum biotin concentration. Another limitation is, that while quota-sampling in the Nutrihealth study provided a good study sample for the vulnerable elderly population, this study was not designed in a way to detailly address another very important population group—pregnant women and those planning pregnancy. Our sample of adults provided interesting insight about this group, but the study sample size limited our ability for deeper analyses of sub-groups. Furthermore, the Nutrihealth study did not address folate deficiency in adolescents and children. It would make sense to further investigate the abovementioned groups, particularly in the context of different supplementation practices.

## 5. Conclusions

For the first time, daily folate intakes with foods were estimated for the Slovenian general population, with a focus on adolescents, adults, and elderlies. In the latter two groups, the prevalence of folate deficiency was also investigated using serum folate and homocysteine as biomarkers. Prevalence of sub-optimal folate intake was observed in all studied populations; however, the prevalence of folate deficiency was noted to a smaller extent. Folate intake (<300 µg/day) was observed in 59% of adolescents, 58% of adults and 68% of elderlies, while about 12% achieved the WHO recommended level of 400 µg/day. Residential area, education, employment status and BMI were linked with low folate intake in adults; BMI, and sex in adolescents; and sex in elderlies. Major dietary contributors were vegetables and fruit, and cereal products (particularly bread). Vegetables were a much more notable contributor in adults and elderlies, in comparison to adolescents. Contrary, folate contribution was higher from processed foods in adolescents, compared to the elderly population. Considering low serum folate (<7 nmol/L) and high serum homocysteine (>15 nmol/L), folate deficiency was found in 7.6 and 10.5% in adults and elderlies, respectively. We observed a trend of higher proportions of folate deficiency in elderlies, males, those living in rural residential areas, with no university degree, below average family net income, higher body mass index and in smokers. Our study indicated that public health strategies should focus on promoting the consumption of fruit, vegetables, legumes, and whole grain foods, particularly in vulnerable population groups. This would be also beneficial, to assure adequate intake of various other micronutrients. Another option for increasing folate intake in the general population is also the introduction of food fortification, but such decisions need to be taken very carefully, and with consideration of the wider European context, because a considerable proportion of the processed foods in the national food supply is originating from other countries. With current folate intakes, supplementation with folic acid should be additionally encouraged in pregnant women and women who plan pregnancy. Standardized prescription of folate medicines (with costs covered from health insurance) for pregnant women and those planning pregnancy would be very beneficial. Additionally, folic acid supplementation should be also considered in some other vulnerable populations, particularly in elderlies.

## Figures and Tables

**Table 1:** Demographic characteristics of the SI.Menu study sample for all three age cohorts (adolescents: 14–17 years; adults: 18–64 years; elderly: 65–74 years).

Variable		Adolescents *N* (%)	Adults *N* (%)	Elderly *N* (%)
Overall (SI.Menu study)		468 (100)	364 (100)	416 (100)
Age (mean ± SD)		13.4 (2.4)	43.6 (13.8)	68.7 (2.7)
Residential area	rural	270 (57.7)	202 (55.5)	229 (55.1)
	intermediate	76 (16.2)	56 (15.4)	71 (17.1)
	urban	122 (26.1)	106 (29.1)	116 (27.9)
Sex	male	238 (50.9)	173 (47.5)	213 (51.2)
	female	230 (49.1)	191 (52.5)	203 (48.8)
Education	no university degree	n.a.	249 (68.4)	342 (82.2)
	university degree	n.a.	115 (31.6)	74 (17.8)
Financial status	below average	n.a.	118 (38.4)	269 (71.5)
	above average	n.a.	189 (61.6)	107 (28.5)
Employment	employed	n.a.	226 (62.1)	n.a.
	unemployed	n.a.	42 (11.5)	n.a.
	student	n.a.	32 (8.8)	n.a.
	retired	n.a.	64 (17.6)	n.a.
BMI (mean ± SD)		21.0 (4.2)	26.7 (5.2)	28.4 (5.0)
BMI	normal	301 (64.6)	148 (40.7)	108 (26.0)
	overweight and obese	167 (35.7)	216 (59.3)	308 (74.0)
Smoking status	current, occasional, ex-smoker	30 (6.4)	165 (45.3)	185 (44.5)
	non smoker	438 (93.6)	199 (54.7)	231 (55.5)
IPAQ	low level	108 (23.3)	127 (35.3)	137 (33.4)
	moderate level	141 (30.5)	108 (30.0)	133 (32.4)
	high level	214 (46.2)	125 (34.7)	140 (34.2)
Supplement use	folate	1 (0.2)	7 (1.9)	1 (0.2)
	multivitamins	128 (27.5)	133 (36.5)	94 (22.6)
	does not use	339 (72.3)	224 (61.6)	321 (77.2)
Behavioural diet	vegetarian/vegan	12 (2.6)	8 (2.2)	3 (0.7)
	no diet	456 (97.4)	356 (97.8)	413 (99.3)
Medical diet	medical and/or weight loss	13 (2.8)	32 (8.8)	51 (12.3)
	no special diet	455 (97.2)	332 (91.2)	465 (87.7)
Subsample of the Nutrihealth study *			125 (34.3)	155 (37.3)

Notes: n.a. = not applicable. SD = standard deviation; BMI = body mass index; For adults and elderlies normal BMI was considered below 25 kg/m^2^, while sex/age adjusted cut-off points were used for adolescents [49,50]; IPAQ = Physical activity according to International Physical Activity Questionnaire; * Serum folate and homocysteine levels available for sub-group participating in the SI.Menu study (Nutrihealth study sample).

**Table 2:** Estimated population-weighted usual daily folate intake and indicators of folate deficiency (95% CI).

	Adolescents *N* (%)			Adults *N* (%)			Elderly *N* (%)		
	All	Male	Female	All	Male	Female	All	Male	Female
Si.Menu study; *N*(%)	468 (100)	238 (50.85)	230 (49.15)	364 (100)	173 (47.53)	191(52.47)	416 (100)	213 (51.20)	203 (48.80)
Daily folate intake									
Mean (95%CI) (µg /day)	289.8 (277.7–301.9)	308.1 (291.9–324.2)	270.1 (255.0–285.3)	294.6 (283.4–305.8)	311.4 (293.8–329.0)	277.5 (264.2–290.9)	295.5 (263.0–327.9)	278.6 (263.7–293.4)	311.0 (252.7-369.3)
Std. Err.	6.16	8.21	7.71	5.70	8.95	6.78	16.5	7.56	29.64
Median (µg /day)	271.5	301.4	247.5	281.2	297.3	267.2	274.1	280.1	270.9
Mean (95% CI) (µg/per 1000 kcal/day) *	127.7 (122.5–132.9)	119.8 (113.0–126.6)	136.3 (129.1–143.6)	138.4 (133.4–143.4)	132.3 (124.7–140.0)	144.6 (138.4–150.8)	140.7 (128.1–153.2)	125.8 (120.9–130.7)	154.3 (133.2–175.3)
Proportion of population with insufficient daily folate intake **									
<300 µg/day	58.7 (51.0–66.1)	49.9 (39.1–60.7)	68.3 (60.1–75.4)	58.1 (52.2–63.8)	52.9 (44.4–61.1)	63.5 (55.3–70.9)	67.8 (58.7–75.7)	73.5 (62.6–82.1)	62.6 (49.9–73.8)
<400 µg/day	87.9 (84.0–90.9)	84.3 (77.8–89.122.2)	91.8 (86.9–95.0)	87.8 (83.5–91.1)	83.5 (76.4–88.7)	92.1 (86.8–95.4)	87.6 (76.9–93.7)	93.3 (88.3–96.2)	82.4 (63.8–92.5)
Nutrihealth study; *N* (%)				125 (100)	52 (41.6)	73 (58.4)	155 (100)	76 (49.0)	79 (51.0)
Serum folate level									
Mean (95%CI) (nmol/L)				10.6 (9.6–11.7)	10.5 (8.9–12.2)	10.8 (9.5–12.1)	11.4 (10.0–12.9)	11.0 (8.6–13.5)	11.8 (10.3–13.3)
Std. Err.				0.54	0.84	0.66	0.72	1.24	0.78
Median (nmol/L)				10.0	9.0	10.0	10.0	9.0	10.0
Prevalence of low serum folate (%) (95% CI)									
<7 nmol/L				16.6 (10.8–24.6)	16.5 (8.6–29.5)	16.6 (9.5–27.6)	18.5 (13.1–25.4)	22.4 (14.3–33.2)	15.0 (8.7–24.7)
<10 nmol/L				49.0 (39.4–58.6)	50.4 (35.7–65.0)	47.5 (35.8–59.4)	48.6 (40.8–56.5)	51.3 (40.1–62.4)	46.3 (35.6–57.3)
Serum homocysteine level									
Mean (95%CI) (µmol/L)				12.6 (11.9–13.3)	13.6 (12.6–14.6)	14.6 (13.9–15.2)	14.6 (13.9–15.2)	16.1 (15.2–17.0)	13.2 (12.5–13.9)
Std. Err.				0.35	0.50	0.42	0.32	0.47	0.36
Median (µmol/L)				12.1	12.6	15.7	14.2	10.9	13.0
Prevalence of high homocysteine level (%) (95% CI)									
>10 µmol/L				75.3 (66.4–82.4)	88.7 (76.5–95.0)	61.0 (48.6–72.2)	88.9 (82.7–93.0)	96.0 (88.3–98.7)	82.5 (72.5–89.4)
>15 µmol/L				20.5 (13.9–29.1)	26.4 (15.8–40.8)	14.2 (8.0–23.9)	39.9 (32.4–47.8	56.6 (45.2–67.3)	25.0 (16.7–35.7)
Prevalence (%) of folate deficiency using criteria of low serum folate (<7 nmol/L) and high serum homocysteine (15 µmol/L)									
				6.9 (3.5–13.0)	9.7 (4.3–20.4)	3.9 (1.8–12.0)	10.1 (6.3–16.0)	14.5 (8.1–24.4)	5.3 (2.6–14.3)

Notes: Estimated daily folate intake with consideration of regular foods (excluding use of food supplements). * conversion factor for µg/MJ is 0.239; ** Cut-off values for daily folate intake according to national/D-A-CH [29,31] (300 µg) and IOM/WHO (400 µg) [27] recommendations; CI: confidence interval.

**Table 3:** Association between daily intake of dietary folate level (>300 µg/day) and sex, residential area, education, income, employment, smoking status, BMI, IPAQ, dietary pattern.

Variable		Adolescents (10–17 Years Old)		Adults (18–64 Years Old)		Elderly (65–74 Years Old)	
		(>300 µg/day) *n* (%)	Odds Ratio *	(>300 µg/day) *n* (%)	Odds Ratio *	(>300 µg/day) *n* (%)	Odds Ratio *
All		181 (38.7)		140 (38.5)		139 (33.4)	
Sex	Male	102 (42.9)	1	78 (45.1)	1	74 (34.7)	1
	Female	79 (34.4)	0.63 (0.43–0.93)	62 (32.5)	0.44 (0.26–0.75)	65 (32.0)	0.87 (0.54–1.42)
Residential area	Rural	104 (38.5)	1	74 (32.3)	1	20 (8.7)	1
	Intermediate	35 (46.1)	1.37 (0.81–2.32)	23 (32.4)	2.25 (1.79–10.18)	6 (8.5)	1.04 (0.57–1.90)
	Urban	42 (34.4)	0.79 (0.50–1.426)	42 (36.2)	1.51 (1.07–4.24)	9 (7.8)	1.23 (0.73–2.07)
Education	No university degree	n.a.	n.a.	83 (33.3)	1	115 (33.6)	1
	University degree			57 (49.6)	1.93 (1.07–3.47)	24 (32.4)	0.93 (0.50–1.73)
Financial status	Below average	n.a.	n.a.	38 (32.2)	1	89 (33.1)	1
	Above average			82 (43.4)	1.11 (0.62–2.00)	38 (35.5)	1.09 (0.65–1.82)
BMI	Normal	126 (41.9)	1	60 (40.5)	1	34 (31.5)	1
	Overweight/obese	55 (32.9)	0.65 (0.43–0.98)	80 (37.0)	0.71 (0.41–1.21)	105 (34.1)	0.98 (0.58–1.64)
IPAQ	Low intensity	40 (37.0)	1	53 (41.7)	1	42 (30.7)	1
	Moderate	62 (44.0)	1.55 (0.91–2.64)	43 (39.8)	0.83 (0.45–1.53)	47 (35.3)	1.19 (0.70–2.03)
	High intensity	79 (36.9)	1.01 (0.62–1.64)	42 (33.6)	0.56 (0.30–1.03)	50 (35.7)	1.12 (0.66–1.90)
Employment status	Employed	n.a.	n.a.	100 (44.3)	1	n.a.	n.a.
	Unemployed			10 (23.8)	0.29 (0.10–0.76)		
	Student			11 (34.4)	0.73 (0.26–2.06)		
	Retired			19 (29.7)	0.60 (0.28–1.28)		
Smoking status	Non smoker	172 (39.3)	1	76 (38.2)	1	75 (32.5)	1
	Current, occasional, ex-smoker	9 (30.0)	0.57 (0.25–1.30)	64 (38.8)	1.34 (0.79–2.28)	64 (34.6)	1.02 (0.63–1.66)
Medical diet	No diet	176 (38.7)	1	129 (38.9)	1	123 (33.7)	1
	Medical and/or weight loss	5 (38.5)	1.17 (0.37–3.74)	11 (34.4)	0.75 (0.29–1.96)	16 (31.4)	0.95 (0.49–1.84)
Behavioural diet	No diet	180 (39.5)	n.a	136 (38.2)	1	138 (33.4)	1
	Veget./vegan	1 (8.33)		4 (50.0)	1.03 (0.22–4.74)	1 (33.3)	0.92 (0.07–11.40)

Note: n.a. = not applicable. BMI = Body mass index; For adults and elderlies normal BMI was considered bellow 25 kg/m^2^, while sex/age adjusted cut-off points were used for adolescents [49,50]; IPAQ = Physical activity according to International Physical Activity Questionnaire; Logistic regression analysis conducted on samples with excluded missing values (Family net income: *N* = 57 (adults) and 40 (elderly); IPAQ: *n* = 5 (adolescents), 4 (adults), 6 (elderly)); * Cut-off odds ratio for intake of over 300 µg of folate per day; Association was significant with following variables: sex, *p* < 0.05 (adolescents), BMI, *p* < 0.05; sex, *p* < 0.005 (adults), education, *p* < 0.05 (adults).

## Data Availability

The data presented in this study are available on request from the corresponding author.

## Supplementary Materials

The following are available online at https://www.mdpi.com/article/10.3390/nu13113860/s1, Supplementary Table S1. Mean (SD) and model adjusted mean (95% CI) usual daily intake of folate by sociodemographic and lifestyle parameters for the three age cohorts. Models adjusted for daily energy intakes (kcal/day), dietary fruit/vegetables intakes (g/day), dietary bread intakes (g/day). Supplementary Table S2. Relative contribution of selected food categories to usual daily dietary folate intake in different age groups (% of total dietary folate intake). Supplementary Table S3. Sample prevalence of folate deficiency and prevalence adjusted odds ratios (95% CI)-by socio-demographic and lifestyle parameters (*N* = 271).
