# Supplementary material for: Dietary Intake of Folate and Assessment of the Folate Deficiency Prevalence in Slovenia Using Serum Biomarkers
Source: Nutrients. 2021 Oct 28;13(11):3860. doi: 10.3390/nu13113860 (PMC8620305; doi:10.3390/nu13113860)
Supplement: Supplementary file 1 [file nutrients-13-03860-s001.zip › nutrients-1422529-supplementary.pdf]

Supplementary Table S1. Mean (SD) and model adjusted mean (95% CI) usual daily intake of folate by sociodemographic and lifestyle parameters for the three age cohorts. Models adjusted for daily energy intakes (kcal/day), dietary fruit/vegetables intakes (g/day), dietary bread intakes (g/day).

Variable	Level	Adolescents (10–17 Years Old)		Adults (18–64 Years Old)		Elderly (65–74 Years Old)	
		Mean (SD)	Adjusted mean (CI)	Mean (SD)	Adjusted mean (CI)	Mean (SD)	Adjusted mean (CI)
Overall *N* (%)		468 (37.5)		364 (29.2)		416 (33.3)	
Sex	Male	300.5 (120.8)	278.7 (267.9–289.5)^{a}	304.3 (103.4)	291.1 (277.5–304.7)	277.7 (104.0)	269.7 (257.5–282.0)^{a}
	Female	273.6 (94.8)	299.7 (288.4–311.1)^{b}	271.7 (82.7)	294.2 (281.2–307.3)	274.3 (89.7)	289.5 (276.9–302.1)^{b}
Residential area	Rural	287.3 (117.0)	290.7 (280.9–300.5)	282.0 (93.4)	280.0 (268.0–292.1)^{b}	273.8 (96.2)	272.2 (261.1–283.3)
	Intermediate	291.1 (88.8)	283.2 (264.2-302.2)	299.2 (96.8)	319.4 (296.8–342.0)^{a}	276.6 (109.1)	290.3 (271.0–309.7)
	Urban	284.8 (104.8)	287.6 (273.0–302.2)	290.8 (96.8)	301.0 (285.4–316.5)^{ab}	280.0 (91.9)	286.6 (270.6–302.5)
Education	No university degree	n.a.	n.a.	275.1 (86.6)	285.4 (274.6–296.3)^{a}	274.2 (95.6)	278.0 (269.0–287.0
	University degree			313.4 (105.2)	309.1 (292.0–326.1)^{b}	284.7 (104.2)	2.85.8 (264.9–306.8)
Family net income	Below average	n.a.	n.a.	271.7 (97.2)	293.4 (278.0–308.9)	274.8 (96.8)	281.9 (272.2–291.6)
	Above average			299.9 (92.6)	292.3 (280.5–304.0)	286.6 (102.9)	273.1 (257.2–289.1)
Employment	Employed	n.a.	n.a.	303.2 (95.5)	304.6 (293.0–316.2)^{A}	n.a.	n.a.
	Unemployed			240.5 (84.1)	263.4 (235.2–291.6)^{B}		
	Student			292.4 (98.9)	285.2 (250.9–319.6)^{AB}		
	Retired			258.5 (78.5)	273.4 (251.9–294.9)^{B}		
Smoking status	Current, occasional ex-smoker	262.1 (113.3)	266.0 (236.6-295.5)	281.6 (92.7)	292.4 (279.4-305.4)	278.1 (104.1)	277.4 (264.8-290.0)
	No smoker	289.0 (109.2)	290.4 (282.6-298.1)	291.8 (95.8)	293.0 (280.6-305.4)	274.4 (91.4)	281.1 (269.5-292.7)
BMI	Normal	295.2 (118.3))	293.5 (284.1–302.8)^{A}	291.7 (98.6)	306.4 (291.5–321.3)^{a}	266.3 (83.0)	271.9 (255.1–288.6)
	Overweight and obese	273.0 (90.3)	280.3 (267.7–292.8)^{B}	284.1 (91.5)	284.6 (273.3–295.8)^{b}	279.4 (101.6)	281.9 (272.5–291.2)
IPAQ	Low intensity	294.6 (103.4)	286.2 (270.5–301.9)	294.0 (95.3)	301.4 (286.6–316.2)	277.0 (112.5)	280.9 (267.0–294.7)
	Moderate	287.7 (110.2)	296.0 (282.3–309.7)	281.8 (89.4)	287.4 (271.6–303.2)	274.4 (88.2)	279.8 (265.6–294.1)
	High intensity	285.2 (112.8)	285.2 (274.1–296.3)	285.0 (98.8)	288.4 (273.3–303.6)	277.9 (91.2)	277.4 (263.3–291.4)
Special diet	No special diet	286.8 (108.5)	287.7 (280.2-295.3)	288.0 (102.4)	291.8 (282.7-300.9)	276.3 (99.5)	280.0 (271.3-288.6)
	Medical and/or weight loss	300.2 (144.8)	322.6 (278.4-366.9)	279.7 (102.4)	302.3 (272.3-332.3)	273.8 (79.4)	275.4 (252.6-298.2)
Behavioural diet	No diet	289.6 (109.4)	289.2 (281.6-296.7)	286.7 (95.0)	292.5 (282.7-300.8)	276.0 (97.1)	279.2 (271.2-287.3)
	Veget./vegan	199.7 (72.5)	266.9 (212.8-321.0)	309.9 (65.6)	305.0 (242.7-367.4)	275.0 (127.1)	299.6 (252.6-298.2)

Note: Body mass index (BMI) was considered as normal below 25 kg/m^2, except for adolescents, where sex/age adjusted cut-off points [22,23] were used. Linear regression analysis conducted on samples with excluded missing values (Family net income: $n = 57$ (adults) and 40 (elderly); International Physical Activity Questionnaire (IPAQ): $n = 5$ (adolescents), 4 (adults), 6 (elderly); Difference in marginal linear predictions per different socio-demographic and other individual related characteristics within age categories: $p < 0.001$ energy (adolescents), $p = 0.005$ intake of fruits and vegetables (adolescents), $p < 0.01$ sex (adolescents), $p = 0.1$ BMI (adolescents); $p < 0.001$ energy (adults), $p < 0.01$ intake of fruits and vegetables (adults), $p < 0.01$ residential area (adults), $p < 0.05$ BMI (adults), $p < 0.05$ education (adults), $p < 0.05$ employment (adults); $p < 0.001$ energy (elderly), $p < 0.001$ intake of fruits and vegetables (elderly), $p < 0.01$ intake of bread (elderly), $p < 0.05$ sex (elderly). The pairwise comparisons are performed using Sidak adjustment; different superscript letters indicate significant differences between groups. Pairwise difference consider significant at $p < 0.1$ indicated by uppercase letters, while lowercase letters indicate difference at $p < 0.05$.

Supplementary Table S2. Relative contribution of selected food categories to usual daily dietary folate intake in different age groups (% of total dietary folate intake).

Food category	Food category contribution in total daily dietary folate intake (%)		
	**Adolescents (10-17 years)**	**Adults (18-64 years)**	**Elderly (65-74 years)**
**Fruit and vegetables**	**27.31**	**39.06**	**43.59**
Vegetables	19.92	30.47	33.01
Fruit	7.40	8.59	10.59
Nuts and seeds	1.10	2.22	1.14
Jam and spreads	0.18	0.17	0.29
**Bread and bakery products**	**32.15**	**25.05**	**30.93**
Bread	21.47	21.51	23.94
White bread	*16.69*	16.03	*16.04*
Brown bread	*4.77*	5.48	*7.90*
Biscuits	6.16	3.50	3.23
Cakes, muffins, and pastry	4.52	3.08	3.76
**Cereal and cereal products**	**12.00**	**8.17**	**2.40**
Breakfast cereals	10.46	5.05	1.88
Unprocessed cereals		0.50	0.01
Other cereal products	*1.54*	2.61	0.51
**Convenience foods**	**2.60**	**2.40**	**2.90**
**Snack foods**	**1.21**	**0.76**	**0.35**
**Meat and meat products**	**5.24**	**6.65**	**5.87**
Processed meat	2.32	2.39	1.49
Unprocessed meat	2.92	4.26	4.38
**Milk and milk products**	**4.87**	**5.58**	**4.44**
**Other**	**14.62**	**12.33**	**9.52**

Supplementary Table S3. Sample prevalence of folate deficiency and prevalence adjusted odds ratios (95% CI)-by socio-demographic and lifestyle parameters (*N*=271).

Variable		Folate deficiency		
		n	**Prevalence *n* (%)**	**Odds Ratio (CI)**
Overall	Level	271 (100)	25 (9.2)	
Age	Adults	118 (43.5)	9 (7.6)	1
	Elderly	153 (56.5)	16 (10.5)	1.22 (0.45-3.29)
Sex	Male	127 (46.9)	17 (13.4)	1
	Female	144 (53.1)	8 (5.6)	0.62 (0.22–1.79)
Residential area	Rural	143 (52.8)	17 (11.9)	1
	Intermediate	41 (15.1)	3 (7.3)	0.92 (0.23–3.64)
	Urban	87 (32.1)	5 (5.8)	0.59 (0.19–1.86)
Education	No university degree	193 (71.2)	21 (10.9)	1
	University degree	78 (28.8)	4 (5.1)	0.73 (0.20-2.61)
Financial status	Below average	136 (53.5)	14 (10.3)	1
	Above average	118 (46.5)	7 (5.9)	0.54 (0.18-1.62)
Employment	Employed	76 (28.0)	2 (2.6)	n.a.
	Unemployed	9 (3.3)	3 (25.0)	
	Student	6 (3.4)	0 (0)	
	Retired	177 (65.3)	20 (11.3)	
BMI	Normal	92 (34.0)	9 (9.8)	1
	Overweight and obese	179 (66.0)	16 (8.9)	0.59 (0.21–1.68)
IPAQ	Low intensity	87 (32.2)	7 (8.1)	1
	Moderate	88 (32.6)	9 (10.2)	1.94 (0.58–6.45)
	High intensity	95 (35.2)	3 (9.5)	1.39 (0.41–4.68)
Smoking status	No smoker	119 (43.9)	11 (7.2)	1
	Current, occasional ex-smoker	152 (56.1)	14 (11.8)	1.40 (0.53-3.66)
Folate intake	≤300 (μg/day)	174 (64.2)	15 (8.6)	1
	>300 (μg/day)	97 (35.8)	10 (10.3)	0.79 (0.26-2.43)
Special diet	No special diet	245 (90.4)	25 (10.2)	n.a.
	Medical and/or weight loss	26 (9.6)	0 (0)	
Behavioral diet	No diet	267 (98.5)	25 (9.36)	n.a.
	Vegetarian / vegan	4 (1.5)	0 (0)	

Notes: Folate deficiency with consideration of combined criteria of low serum folate (<7 nmol/L) and high serum homocysteine (>15 μmol/L) concentration. n.a. indicates variables that were not included in the model. Body mass index (BMI) was considered as normal below 25 kg/m^2, except for adolescents, where sex/age adjusted cut-off points [22,23] were used. Analysis conducted on samples with excluded missing values (Family net income, n = 17; International Physical Activity Questionnaire (IPAQ), n=1; Education, n=1; Employment, n=1). Model is adjusted for daily energy intake (Kcal/day), which was not significant predictor of folate deficiency (OR: 1.0008, p = 0.127).
